# Assessment of oppositional defiant disorder and oppositional behavior in children and adolescents with Down syndrome

**DOI:** 10.3389/fpsyt.2022.1062201

**Published:** 2023-01-16

**Authors:** Elisa Fucà, Flavia Cirillo, Laura Celestini, Paolo Alfieri, Diletta Valentini, Floriana Costanzo, Stefano Vicari

**Affiliations:** ^1^Child and Adolescent Neuropsychiatry Unit, Department of Neuroscience, Bambino Gesù Children’s Hospital, IRCCS, Rome, Italy; ^2^Pediatric Unit, Pediatric Emergency Department (DEA), Bambino Gesù Children’s Hospital, IRCCS, Rome, Italy; ^3^Department of Life Science and Public Health, Catholic University of the Sacred Heart, Rome, Italy

**Keywords:** psychopathology, SNAP-IV, Conners’ Parent Rating Scales, CBCL, K-SADS, trisomy 21 (Down syndrome)

## Abstract

**Introduction:**

Children and adolescents with intellectual disability (ID) exhibit higher rates of oppositional defiant disorder (ODD) than typically developing (TD) peers. However, studies focusing on the investigation of ODD prevalence in youth with Down syndrome (DS) are still limited.

**Methods:**

The current study aimed to investigate the prevalence of ODD clinical and subclinical symptoms in a group of 101 youth with DS (63 boys, 38 girls) ranging in age from 6 to 18 years. Moreover, the prevalence of ODD symptoms, as detected by means of three parent-report questionnaires, was compared with that detected by a semi-structured psychopathological interview, namely, the Schedule for Affective Disorders and Schizophrenia for School Aged Children Present and Lifetime (K-SADS) Version Diagnostic and Statistical Manual of Mental Disorders-5 (DSM-5).

**Results:**

We found that 17% of participants met diagnostic criteria for ODD on the K-SADS, whereas 24% exhibited subclinical symptoms. Results also suggest good specificity of Swanson, Nolan, and Pelham-IV Rating Scale (SNAP-IV), Conners’ Parent Rating Scales Long Version (CPRS) and Child Behavior Checklist (CBCL) in detecting ODD symptoms. The investigation of the agreement in the prevalence rates of clinical and subclinical symptoms of ODD between K-SADS and the parent-report questionnaires indicated CPRS as the parent-report questionnaire with the best agreement with K-SADS.

**Discussion:**

This study provides support for the use of parent-report questionnaires to assess ODD symptoms in children and adolescents with DS by evaluating their levels of agreement with a semi-structured psychopathological interview. In particular, our results suggest that CPRS could be considered a suitable screening tool for ODD clinical and subclinical symptoms in youth with DS.

## 1. Introduction

Oppositional defiant disorder (ODD) has been defined as “a pattern of angry/irritable mood, argumentative/defiant behavior, or vindictiveness lasting at least 6 months,” characterized by irritable mood (e.g., “often loses temper”), argumentative/defiant behavior (e.g., “Often deliberately annoys others”) and/or vindictiveness (1). For children younger than 5 years, the behavior should occur on most days for a period of at least 6 months unless otherwise noted, whereas for individuals 5 years or older, the behavior should occur at least once per week for at least 6 months, unless otherwise noted (1). Although the symptoms may be limited to one setting–most frequently the home–in severe cases the symptoms are present in multiple settings. Since the pervasiveness of symptoms is an indicator of the severity of ODD, the clinical assessment should then take into account multiple settings and relationships (1).

The estimated prevalence of ODD in general population ranges from 0.2 to 11%, with an average prevalence estimate of around 3.3% (1–4). ODD is one of the most frequent disorders in early childhood, with prevalence rates of 4.0–16.8%; the pooled prevalence is 3.6% up to age 18 (5–8), while clinic samples studies suggest a range of 28–65% of children meeting diagnostic criteria (9). Subclinical conditions can be identified in cases in which individual has ODD symptoms but does not fully meet the criteria for the diagnosis, for instance, when temporal criteria are not fully met or when the individual exhibits less than the four symptoms required to meet diagnosis. A gender disparity in middle childhood of 1.59:1 for boys to girls has been reported (10); however, girls seem to exhibit higher rates of the disorder until equal prevalence between genders by adolescence (9). ODD is generally conceived as a childhood condition; however, accumulating evidence suggests ODD can persist in adulthood (11, 12). As concerns the male-to-female ratio in adulthood, comparable levels of ODD symptoms between men and women have been reported (13, 14).

The presence of ODD in children is associated with higher risk for a wide range of future psychopathology in later adolescence and adulthood; moreover, ODD determines impairment in multiple domains, such as social and academic functioning (1, 15–18). Impairment caused by ODD goes beyond individual impact: ODD can cause significant distress to caregivers (19) and affect parents’ abilities of emotion regulation (20); then, it is not surprising that mothers of children with ODD symptoms exhibit lower levels of quality of life (21). Considering such a significant impact, proper recognition and timely intervention are crucial.

Oppositional defiant disorder seems to be more frequent in children with intellectual disability (ID) than typically developing (TD) youth. The relative risk ratios of children with ID in comparison to TD children ranges from 1.60:1 to 1.70:1 (22, 23). Emerson and Hatton (24) reported prevalence rate of 11.1% in individuals with ID compared with 2.3% in TD. A subsequent research reported prevalence estimates ranging from 34.7% at 7 years of age to 44.9% at 5 years of age in a group of children with ID; differently from what reported in general population, no gender differences emerged (25). Another study reported a prevalence of 21.6% in school-aged children with mild ID (26); finally, a more recent study found prevalence of 8.4% in children with ID and 3% for TD (27). One of the possible reasons explaining such variability in estimated prevalence of ODD is linked with differences in the methods used for the clinical assessment. The lack of standardized instruments for the assessment of ODD symptoms in individuals with ID hinders not only the settlement of valid diagnostic cut-offs but also the investigation on the prevalence rates (27).

Among developmental disabilities, relatively poor attention has been devoted to the investigation of ODD in individuals with Down syndrome (DS), which is the most frequent genetic cause of ID (28). Although it has been widely recognized that children with DS frequently exhibit behavioral problems (29–33), studies specifically focusing on the investigation of ODD prevalence are limited and do not show consistent results. A cross sectional study on a sample of 100 children and adolescents with DS reported a prevalence of 8% in DS and of 14% in controls (34). A more recent research involving 97 participants with DS aged 1–18 years found the 26% of the sample screened positive for ODD symptoms (35).

The methodology employed to assess ODD in DS varied between the studies. For instance, one study (34) used the Arabic version of Mini International Neuropsychiatric Interview for Children (36) and the Disruptive Behavior Disorder Rating Scale (37). The Mini International Neuropsychiatric Interview for Children is a short diagnostic interview fully structured to allow administration by non-specialized interviewers (38). The instrument exhibited good specificity for all diagnoses (range: 0.72–0.97) as well as high inter-rater reliability, with kappa coefficients ranging from 0.88 to 1.0 (38). However, some limitations in the validation studies have been highlighted, such as small sample size (36). The Disruptive Behavior Disorder Rating Scale consists of 41 Diagnostic and Statistical Manual of Mental Disorders-IV (DSM-IV) items; with 18 items related to attention deficit/hyperactivity disorder (ADHD), 8 items related to ODD, and 15 items to conduct disorder (37). The instrument has good internal coherence, with alphas values ranging from good (0.78) to excellent (0.96) (37). To the best of our knowledge, the psychometric properties of the instrument were not investigated in Italian population. Another research employed the Child Behavior Checklist (CBCL), an empirically based checklist of social competence and behavioral problems, to assess the presence of ODD symptoms in youth with DS (35). Normative populations indicated reliable internal consistency values of 0.78–0.97 for the full scales (39), whereas values of inter-parent agreement range from 0.26 to 0.78 (40). In another study (41), ODD diagnosis in youth with DS based only on the DSM (1, 42), without the support of specific instruments. Gothelf et al. (43) involved a sample of adolescents with different forms of ID, including individuals with DS, and employed the Schedule for Affective Disorders and Schizophrenia for School-Aged Children, Present and Lifetime (K-SADS) (44) to support the psychiatric diagnosis. On the other hand, although not specifically focusing on ODD diagnosis, some studies have investigated disruptive behaviors more broadly in youth with DS, using the Aberrant Behavior Checklist (45–47). Its reliability and validity was supported by the authors (45, 48) and independent researchers (49, 50). Moreover, a good criterion validity also in a population of individuals with DS has been established (51).

Since investigations on ODD in DS are still rare, a few issues remain to be clarified, namely: (i) whether gender differences in the occurrence of ODD exist in youth with DS; (ii) what is the occurrence of subclinical ODD in youth with DS; (iii) what is the concordance between clinical examination and parent-report assessment for detection of ODD. This aspect is particularly relevant since several instruments employed for psychopathological evaluation are not specifically developed for population with ID.

The current study had two main objectives:

• The investigation of the prevalence of ODD diagnosis and subclinical ODD in a group of youth with DS.
• The comparison of the ODD symptoms detection of three widely used parent-report questionnaires [i.e., the CBCL, the Swanson, Nolan, and Pelham–IV Rating Scale (SNAP-IV) and the Conners’ Parent Rating Scales Long Version (CPRS), Revised] vs. a semi-structured psychopathological interview, namely, the K-SADS, that is regarded as the criterion standard for child psychiatric diagnoses (52–54) and it has been used also for psychopathological assessment of children and adolescents with borderline intelligence quotient (IQ) (55) and with ID (26, 56, 57).

Considering past research indicating prevalence estimates of ODD symptoms in DS ranging from 8 to 26%, we hypothesized that prevalence rate of ODD would fell within this range in the current study. Moreover, basing on literature supporting the use of the three parent-report instruments for the detection of ODD symptoms in general population and in other neurodevelopmental disorders (58–61), we hypothesized that these instruments were equally accurate in identifying ODD in children and adolescents with DS.

## 2. Materials and methods

### 2.1. Participants

One-hundred and one children with DS (63 boys, 38 girls) ranging in age from 6 to 18 years (mean 8.97 ± 2.24 years) were included in the study. The mean IQ was 54.8 ± 6.65. Selection criteria included, besides the diagnosis of DS based on the analysis of the karyotype, the age ranging between 6 and 18 years. Exclusion criteria were as follows: age < 6 and > 18 years; language barrier hampering questionnaire compilation by parents (the Italian version of the questionnaires was administered). All participants underwent a child psychiatric and neuropsychological examination conducted by experienced developmental neuropsychiatrists and neuropsychologists.

### 2.2. Procedure

This is a cross-sectional study; data were retrospectively collected from a file review of patients with DS referred for a clinical evaluation at the Child and Adolescent Neuropsychiatry Unit of the Bambino Gesù Children’s Hospital in Rome. The clinical evaluation of children and adolescents with DS consisted in a neuropsychiatric, neuropsychological, and psychopathological/behavioral assessment performed by a team made of a child neuropsychiatrist and clinical psychologists and neuropsychologists with clinical expertise on DS. The clinical evaluation also included the administration of parent-report questionnaires, which were filled out by the parents while the children underwent neuropsychological or behavioral evaluation. Parents received precise instructions regarding filling out the questionnaires. In accordance with the objectives of the study, only participants whose information was obtained from mothers were included. From the original database including 268 records, 167 files were excluded because they did not meet inclusion criteria or because of missing data; therefore a total of 101 participants were included in the study.

Due to the retrospective design, data were collected from the hospital records and clinic charts and the de-identified data were analyzed. All parents signed a written informed consent for data use for research purposes and a privacy statement that ensures that data will be kept confidential. For the current project, all subjects meeting specified criteria as described above were selected from a database. The study was conducted according to the guidelines of the Declaration of Helsinki and it was approved by the local Ethics Committee (process number 2202_OPBG_2020).

### 2.3. Measures

Cognitive development was tested by the Leiter-3 (62),which provides a non-verbal measure of intelligence and assesses the ability to reason by analogy, matching and perceptual reasoning, irrespective of language, and formal schooling for individuals ages 3–70.

Schedule for Affective Disorders and Schizophrenia for School Aged Children Present and Lifetime Version DSM-5. K-SADS is a semi-structured psychopathological interview that investigates the possible presence of psychopathological disorders according to DSM-5 (44). The K–SADS has a three-point scale, where 1 = symptom is absent, 2 = symptom is present at a subclinical level, and 3 = symptom is severe and frequent enough to be at or above threshold. The K-SADS, as proposed in the instrument manual by Kaufman et al. (44) provides as a source of information not only the child/adolescent but also the parent(s). For some particular cases (i.e., ID), the parent is considered the main source of information with respect to the child. If general symptoms emerge in the screening interview, questions from the appropriate supplement are used to verify the diagnosis. We considered subthreshold symptoms to be subclinical psychopathology.

Swanson, Nolan, and Pelham–IV Rating Scale (SNAP-IV) is a screening tool for ADHD and includes the items according to DSM–IV and DSM-5 (1, 63, 64). The items are designed to distinguish between different symptom presentations of ADHD, namely, inattentive (items 1–9), hyperactive-impulsive (items 10–18), and combined (both inattentive and hyperactive/impulsive). The questionnaire also includes questions about ODD (items 19–26). For each item, respondents select one of four response options (0 “Not at all,” 1 “Just a little,” 2 “Quite a bit,” or 3 “Very much”). Subscale and total scores are calculated as an average score across relevant items. In the current study, scores from the ODD scale were considered.

Conners’ Parent Rating Scales Long Version, Revised. The CPRS (65) is a widely used tool for the screening of ADHD and related symptoms. The instrument includes 80 items and it is composed of 14 different scales, namely: oppositional; inattention; hyperactivity; anxiety; perfectionism; social problems; psychosomatic problems; ADHD index; CGI: restlessness; CGI: emotional instability; CGI: total; DSM-IV: inattention; DSM-IV: hyperactivity/impulsivity; DSM-IV: total. Items are rated on a four-point rating scale ranging from 0 (not true at all) to 3 (very much true). The instrument generates a *T*-score for each subscale. The cutoff for *T*-scores for clinical significance is > 70 (very elevated) and *T*-scores from 60 to 70 are considered as high averages or elevated. In the current study, scores from the oppositional scale were considered.

Child Behavior Checklist 6–18. The CBCL (66) is a 113-item parent-report instrument designed to assess behavior and emotion related problems in children. It generates eight syndrome scales (Anxious/depressed, Withdrawn/depressed, Somatic complaints, Social problems, Thought problems, Attention problems, Rule-breaking behaviors, and Aggressive behaviors), and two broad-band scales (Internalizing problems and Externalizing problems). The sum of all the items generates the “Total Problem” scale. The CBCL also embraces six DSM-Oriented Scales (Affective, Anxiety, Somatic, ADHD, Oppositional defiant problems, and Conduct problems). Parents are asked to rate each behavior’s frequency on a three-point Likert scale (0, not true; 1, somewhat or sometimes true; 2, very true or often true). In the current study, scores from the oppositional defiant problems scale were considered.

### 2.4. Statistical analyses

Qualitative variables were presented as percentages, and chi-square test was used for group comparisons. The analysis of covariance (ANCOVA) was used to compare the means for each screening tool between individuals with and without ODD (assessed with the K-SADS; ODD + and ODD − groups, respectively) whilst controlling potential confounding factors (IQ, age, ADHD symptoms as detected by K-SADS). Receiver–operator curve (ROC) analyses were conducted using Statistical Package for Social Science, version 13.0 (IBM Corp., Armonk, NY, USA). For each ROC analysis, we calculated area-under-the-curve (AUC); the critical value for significance for the AUC was set at *p* = 0.05. For each cutoff score provided in literature, we computed its sensitivity and specificity. AUC values > 0.80 are very good, 0.70–0.80 are good, 0.60–0.70 are sufficient, and <0.60 are bad and not useful (67). Hierarchical linear regressions examined convergent and divergent validity of the ODD and ADHD diagnoses as described elsewhere (68). Briefly, diagnostic status, as detected by means of K-SADS, was coded as dichotomous variables (present/absent). The diagnostic category of interest, namely, ODD, was entered as the independent variable in step 1, and ADHD was entered in step 2. Rating scale, e.g., CPRS oppositional scale, was entered as the dependent variable. Separate analyses were conducted for each rating scale. Convergent validity was confirmed if the diagnostic category significantly predicted the dependent variable in step 1. Divergent validity was confirmed if the first diagnostic category still predicted the dependent variable in step 2 and the second diagnostic category did not. Partial correlation analysis was run to investigate the association between K-SADS and scores from parent-report questionnaires whilst controlling for age, sex, IQ, and the presence of ADHD symptoms. The differences of ODD symptoms distribution between the different instruments were analyzed by chi-square analyses. Kappa statistics were used to determine agreement between K-SADS and parent-report questionnaires. Kappa is considered to be slight if <0.2, fair if 0.21–0.40, moderate if 0.41–0.60, substantial if 0.61–0.80, and almost perfect if 0.81–1.00.

## 3. Results

### 3.1. Prevalence of ODD symptoms

**FIGURE 1:**
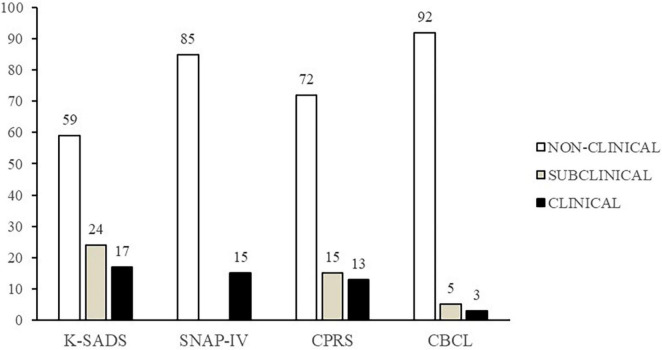
Distribution of non-clinical, subclinical, and clinical scores as detected through Swanson, Nolan, and Pelham–IV Rating Scale (SNAP-IV), Conners’ Parent Rating Scales Long Version (CPRS), Child Behavior Checklist (CBCL), and Schedule for Affective Disorders and Schizophrenia for School Aged Children Present and Lifetime (K-SADS) (%).

K-SADS administration revealed a prevalence of 17% who meet criteria for ODD and of 24% who meet criteria for subclinical ODD. Clinical score rates detected by questionnaires were, respectively: SNAP-IV 15, CPRS 13, and CBCL 3%. As concerns subclinical scores, CPRS and CBCL detected, respectively, 15 and 5% of parent-reported subclinical scores; it must be underlined that SNAP-IV does not produce subclinical scores. Results on the prevalence of ODD symptoms as detected by K-SADS, SNAP-IV, CPRS, and CBCL are summarized in Figure 1. Chi-square analysis failed to detect significant differences between sexes in the prevalence of ODD symptoms as evaluated by K-SADS (fully met ODD criteria: 12% males and 5% females; subclinical ODD: 15% males, 9% females; non-clinical: 37% males and 23% females; *p* = 0.792).

### 3.2. Convergent validity and divergent validity

Compared to participants without ODD symptoms, those who met diagnostic criteria for ODD based on the K-SADS scored significantly higher on all the questionnaires considered. Indeed, ANCOVA analysis revealed significant differences between groups on SNAP-IV scores, *F* (1,96) = 8.619, *p* = 0.004, η*p*^2^ = 0.08 (*M* = 10, SD = 5.9 for the ODD + group and *M* = 5.81, SD = 4.76 for the ODD − group). Significant differences between groups emerged also for CPRS scores, *F* (1,96) = 7.519, *p* = 0.007, η*p*^2^ = 0.07 (*M* = 56.63, SD = 5.77 for the ODD + group and *M* = 53.9, SD = 4.65 for the ODD − group), as well as for CBCL scores *F* (1,96) = 5.825, *p* = 0.02, η*p*^2^ = 0.06 (*M* = 56.63, SD = 5.76 for the ODD + group and *M* = 53.9, SD = 4.65 for the ODD − group).

Given the high frequency of co-occurrence of ODD and ADHD (69), we explored divergent validity of ODD with reference to ADHD diagnosis. Hierarchical regression analysis revealed that the presence of an ODD diagnosis significantly predicted symptom ratings of ODD as reported by parents for all the questionnaires considered in the current study. Specifically, ODD diagnosis (as detected by K-SADS) was entered in the first step of the regression equation predicting SNAP-IV scores and was significant (Table 1), accounting for the 13% of variance. ADHD diagnosis accounted for no significant additional variance in SNAP-IV, beyond that accounted for by ODD diagnosis (Table 1). Then, ODD diagnosis (as detected by K-SADS) was entered in the first step of the regression equation predicting CPRS scores and was significant (Table 1), accounting for the 10% of variance. ADHD diagnosis accounted for no significant additional variance in SNAP-IV, beyond that accounted for by ODD diagnosis (Table 1); the total model accounted for the 12% of variance. Finally, ODD diagnosis significantly predicted CBCL scores, accounting for the 6% of variance. ADHD diagnosis accounted for no significant additional variance, beyond that accounted for by ODD diagnosis (Table 1).

**TABLE 1:** Convergent and divergent validity of ODD and ADHD diagnoses.

		SNAP-IV			CPRS			CBCL		
Model	Diagnostic category	B	SE	Δ R^2^	B	SE	Δ R^2^	B	SE	Δ R^2^
1	ODD	0.391	1.088	0.153*	0.330	2.475	0.109*	0.256	1.039	0.065*
2	ODD	0.345	1.106	0.033	0.286	2.552	0.024	0.289	1.078	0.013
	ADHD	0.186	1.875		0.162	3.35		−0.121	1.415	

ODD, oppositional defiant disorder; ADHD, attention deficit/hyperactivity disorder; SNAP-IV, Swanson, Nolan, and Pelham–IV Rating Scale; CPRS, Conners’ Parent Rating Scales Long Version; CBCL, Child Behavior Checklist; SE, standard error. *p < 0.05.

Significant correlations emerged between K-SADS and SNAP-IV (*r* = 0.386; *p* < 0.001), CPRS (*r* = 0.401; *p* < 0.001), and CBCL (*r* = 0.426; *p* < 0.001) scores.

### 3.3. Agreement between K-SADS and questionnaires

Chi-square test was used to investigate if the distribution of clinical, subclinical, and non-clinical rates as detected by K-SADS differed from those emerging by the three parent-report questionnaires identified in the current study. Since SNAP-IV does not produce subclinical scores, K-SADS clinical and subclinical rates were merged in the comparison between these two instruments. Significant differences emerged between the score distribution as detected by K-SADS and those detected by SNAP-IV and CBCL (χ^2^ = 15.61, *p* < 0.001 and χ^2^ = 27.99, *p* < 0.001, respectively), whereas differences between K-SADS and CPRS did not emerge (*p* = 0.177).

ROC was generated for each instrument considered. As concerns SNAP-IV, AUC was 0.707 (CI at 95%: 0.602–0.812), indicating good accuracy of this tool for ODD detection in DS. Sensitivity and specificity considering the cut-off points provided in the published psychometric studies for Italian population for the ODD scale (i.e., 1.88) were as follows: 19.5–24.4% and 91.7–96.7%, respectively. Given the low sensitivity values, we identified the cut-off points to maximize test efficiency as 1.125 (Sensitivity: 60%; Specificity: 76.7%). As concerns CPRS, AUC was 0.703 (CI at 95%: 0.689–0.717), indicating good accuracy of this tool for ODD detection in DS. Sensitivity and specificity considering the cut-off points provided in literature were as follows: 52.8–45.3% and 76.6–78.6%, respectively. Given the low sensitivity values, we identified the cut-off points to maximize test efficiency as a *T*-score of 56.5 (Sensitivity: 62.4%; Specificity: 70.8%). As concerns CBCL, AUC was 0.657 (CI at 95%: 0.643–0.672), indicating sufficient accuracy of this tool for ODD detection in DS. Sensitivity and specificity considering the cut-off points provided in literature were as follows: 14.7–11.9% and 93.8–97.8%, respectively. Given the very low sensitivity values, we identified the cut-off points to maximize test efficiency as a *T*-score of 54 (Sensitivity: 50.4–69.3%; Specificity: 53.5%).

Finally, kappa index showed an agreement between the K-SADS and the SNAP-IV of 0.178; the concordance with CPRS was 0.268, whereas the lowest level of concordance emerged with CBCL was 0.047.

## 4. Discussion

The first aim of the present study was to provide a prevalence estimation of ODD in a large group of children and adolescents with DS. To this aim, we employed a semi-structured psychopathological interview (K-SADS). We found 17% of participants met diagnostic criteria for ODD on the K-SADS, whereas 24% exhibited subclinical symptoms; this prevalence is similar to that reported for children with ID (24). However, our results differ from what has been previously found in youth with DS. Indeed, previous studies reported 8 and 26% of youth screened positive for ODD (34, 35). These differences could be explained, at least in part, by methodological dissimilarities between previous research and the current study, since the instruments and methods used to support ODD diagnosis varied. However, differences in prevalence emerges also considering the percentage of ODD clinical and subclinical scores as detected by CBCL in comparison with other research using the same instrument. Indeed, in the current study CBCL administration revealed only 3% of clinical scores and 5% of subclinical scores. This is different from what has been previously reported in a study that identified a prevalence of 26% supported by CBCL administration in 97 participants with DS, aged 1–18 years (35). Of note, the age range of participants varied between studies: toddlers and preschoolers were excluded from the current study. Since ODD symptoms seem to decrease with age (70)–as also observed by Marino et al. (35)–it is supposable that the higher age of participants included in the current study contributes explaining the lower observed prevalence.

Consistently with previous findings on ID (25), sex differences in ODD symptom distribution did not emerge, indicating that boys and girls with DS meet ODD diagnostic criteria at similar rates. However, since ODD symptoms seem to reach an equal sex distribution in typically developing population only in adolescence (9), future studies should focus on the prevalence of ODD symptoms across different age range, distinguishing between school-aged children and adolescents with DS.

The second aim of this study was to compare the ODD symptoms detection of SNAP-IV, CPRS, and CBCL with ODD symptoms as detected by K-SADS in children and adolescents with DS. These parent-report questionnaires (i.e., SNAP-IV, CPRS, and CBCL) include scales for the identification of ODD symptoms and they have been previously used in population with DS (35, 71, 72). Similar percentages of clinical scores emerged after SNAP-IV and CPRS administration; whereas clinical scores detected by CBCL were lower (3%). As concerns subclinical scores, CPRS identified 15% of participants in the subclinical range, whereas CBCL only 5%. Analysis of the convergent validity suggested that all the questionnaires are effective in identifying children with DS who met the diagnostic criteria for ODD on the K-SADS. Indeed, after controlling for potential confounding factors, namely, IQ, age, and ADHD symptoms, participants who screened positive for ODD on K-SADS exhibited significantly higher scores than children who did not at all of the questionnaires considered.

However, some dissimilarities between instruments emerged: differences in CBCL scores between participants who received an ODD diagnosis and participants who did not were less pronounced in comparison with the other questionnaires. As concerns divergent validity, results from hierarchical regression suggested that all of the questionnaires are able to discriminate well between ODD and ADHD symptoms, which frequently co-occur. Indeed, ADHD diagnosis did not predict symptoms of ODD in any of the questionnaires considered. Altogether, these findings suggest good specificity of SNAP-IV, CPRS, and CBCL in detecting ODD symptoms.

The next step was the investigation of the agreement in the prevalence rates of clinical and subclinical symptoms of ODD between K-SADS and the parent-report questionnaires. Of note, no differences emerged in the comparison between K-SADS and CPRS, suggesting a good level of accordance between the instruments. This result was further confirmed by kappa index that revealed the highest level of concordance between K-SADS and CPRS. As concerns SNAP-IV, significant differences emerged in the distribution of clinical and non-clinical scores; moreover, kappa index revealed slight agreement with K-SADS. Similarly, the distribution of clinical, subclinical and non-clinical scores significantly differed between CBCL and K-SADS; in addition, kappa statistics revealed that CBCL had the lowest agreement with K-SADS in comparison with the other questionnaires. Altogether, these results seem to indicate CPRS as the parent-report questionnaire with the best agreement with K-SADS. It must be underlined that kappa values were substantially low for all of the instruments included in the current study. A possible explanation may be linked to the different kind of informant considered by the different instruments. Indeed, the K-SADS is clinician-mediated whereas questionnaires are entirely parent-report. However, despite some limitations, the usefulness of parent-report questionnaires in psychological and psychopathological screening has been recognized in clinical practice, including the clinical assessment of children with DS (73, 74). Thus, the current study provides indication of CPRS as a more accurate tool, in comparison with the other parent-report questionnaires considered, to screen for ODD in youth with DS.

ROC analysis revealed that the parent-report questionnaires could be considered sufficiently accurate in screening for ODD symptoms in DS population, as indicated by the AUC > 0.60 in all cases. However, it emerged that the SNAP-IV cut-off provided in literature for ODD scale was poorly sensitive for children and adolescents with DS; it would therefore be appropriate to lower the cut-off to guarantee better sensitivity of the instrument for this population. This finding is not surprising if we consider there is insufficient evidence about the psychometric properties of SNAP-IV in clinic-referred populations (75), especially for ODD. Similarly, cut-off provided in literature for CBCL ODD scale exhibited very high specificity, but an extremely low sensitivity. Also in this case it would be recommendable to considerably lower the cut-off to provide sufficient sensitivity for the detection of ODD symptoms in youth with DS. Of note, low sensitivity emerged also for CPRS, but there was a less pronounced discrepancy between the cut-off provided in literature and that we suggested to reach a sufficient balance between sensitivity and specificity to capture ODD symptoms in DS population. In sum, despite parent report questionnaires revealed to be sufficiently accurate in ODD symptom detection in children and adolescents with DS, they seem poorly sensitive for this population. Therefore, to reduce the risk of false negatives, it could be appropriate to lower the cut-offs of these parent-report instruments. It must be highlighted, however, that CPRS appear to be the instrument with the lowest risk of false negatives among those considered.

Psychopathological assessment in children and adolescents is a complex process that needs gaining lifetime and current information about the individual and his/her functioning across different environments. This process becomes even more demanding when assessing children and adolescents with ID. Indeed, challenges in the interpretation of symptoms can emerge within the assessment process; these challenges are often due to linguistic impairments that frequently occur in individuals with ID and, more specifically, in youth with DS. Diagnostic interviews, such as the K-SADS, can be valuable tools to investigate psychopathological symptoms in children and adolescents, including those with ID. Indeed, the employment of structured or semi-structured diagnostic interview has been found to support diagnostic reliability (76, 77); moreover, patients can experience the diagnostic interview and the relationship with the interviewer as positive and useful (78). However, the administration of interviews is time consuming and there is need for efficient screening instruments for the detection of ODD symptoms in DS. Some informant-based measures to evaluate behavioral problems in children with ID have been developed, such as the Nisonger Child Behavior Rating Form (79), which has been validated also in its Italian version (80), and the Aberrant Behavior Checklist (45). However, these instruments are mainly focused on challenging behaviors rather than on ODD symptoms. The current study investigated the suitability, for DS population, of questionnaires that have been specifically developed to include the assessment of ODD symptoms.

To the best of our knowledge, this is the first study providing support for the use of parent-report questionnaires to assess ODD symptoms in children and adolescents with DS by evaluating their levels of agreement with a semi-structured psychopathological interview. In particular, our results suggest good specificity of all the instruments considered, with CPRS having the best level of agreement with the psychopathological interview among the parent-report questionnaires considered. Therefore, CPRS could be considered as suitable screening tool for ODD symptoms in youth with DS.

It must be recognized that some specificities in the clinical manifestations of ODD in youth with ID, including those with DS, can emerge. Previous evidence suggested that ODD is the same disorder for children with ID as for children with typical cognitive development (25) and that ODD symptoms of DSM-5 may be valid for the assessment of the disorder in children with ID (27). However, a recent study aiming to investigate whether there are differences in the functioning of ODD symptoms between children with and without ID found that two symptoms, namely, “annoys others on purpose” and “argues with adults” seem to be non-invariant (27). Therefore, some clinical features that are frequently associated with DS, such as language deficits, should be accurately taken into account when assessing ODD in this population, especially for symptoms whose expression require a strong verbal component.

The main limitation of the present study is a possible enrollment bias, since participants were selected from a database of patients who underwent a clinical evaluation at a Child and Adolescent Neuropsychiatric service for a screening for neurodevelopmental or psychopathological disorders associated with DS and ID. Further research is required to study the prevalence of ODD symptoms in community samples of individuals with DS. Another limitation of the current study is the lack of a comparison with groups of individuals with other forms of ID; this would allow a more accurate characterization of ODD symptomatology across different syndromes. Finally, given that the present study focused on information provided by mothers, further research is required to investigate potential differences in the use of these instruments between different informants, such as fathers and teachers.

Despite these limitations, our study offers crucial insights into the prevalence of ODD symptoms in youth with DS and provides useful indications for the detection of ODD in this population. We provided evidence supporting the use of CPRS for the identification of ODD symptoms in children and adolescents with DS. This could have significant clinical implications, considering that parent-report questionnaires represent easy-to-use tools to guide clinicians to query symptom areas requiring further assessment. These findings emphasize the importance of properly assessing behavioral problems with specific instruments for different population with neurodevelopmental disorders.

## Data availability statement

The raw data supporting the conclusions of this article will be made available by the authors, without undue reservation.

## Ethics statement

The studies involving human participants were reviewed and approved by the Bambino Gesù Children’s Hospital Ethics Committee. Written informed consent to participate in this study was provided by the participants or their legal guardian/next of kin.

## Author contributions

EF, FCo, and SV: conceptualization, methodology, formal analysis, and writing—original draft preparation; EF, FCi, LC, and DV: investigation. EF and FCi: data curation. EF, FCo, PA, and SV: writing—review and editing. SV: supervision and project administration. All authors have read and agreed to the published version of the manuscript.
